# Reduced concentrations of the B cell cytokine interleukin 38 are associated with cardiovascular disease risk in overweight subjects

**DOI:** 10.1002/eji.201948390

**Published:** 2020-11-19

**Authors:** Dennis M. de Graaf, Martin Jaeger, Inge C. L. van den Munckhof, Rob ter Horst, Kiki Schraa, Jelle Zwaag, Matthijs Kox, Mayumi Fujita, Takeshi Yamauchi, Laura Mercurio, Stefania Madonna, Joost H.W. Rutten, Jacqueline de Graaf, Niels P. Riksen, Frank L. van de Veerdonk, Mihai G. Netea, Leo A.B. Joosten, Charles A. Dinarello

**Affiliations:** ^1^ Department of Medicine University of Colorado Denver Aurora CO USA; ^2^ Department of Internal Medicine and Radboud Institute of Molecular Life Science (RIMLS) Radboud University Medical Center Nijmegen The Netherlands; ^3^ Department of Intensive Care Medicine and Radboud Institute of Molecular Life Science (RIMLS) Radboud University Medical Center Nijmegen The Netherlands; ^4^ Department of Dermatology University of Colorado Denver Aurora CO USA; ^5^ Laboratory of Experimental Immunology IDI‐IRCCS Fondazione Luigi M. Monti Rome Italy

**Keywords:** IL‐38, Cardiovascular disease risk, B cells, Inflammation, Obesity

## Abstract

The IL‐1 family member IL‐38 (IL1F10) suppresses inflammatory and autoimmune conditions. Here, we report that plasma concentrations of IL‐38 in 288 healthy Europeans correlate positively with circulating memory B cells and plasmablasts. IL‐38 correlated negatively with age (*p* = 0.02) and was stable in 48 subjects for 1 year. In comparison with primary keratinocytes, *IL1F10* expression in CD19^+^ B cells from PBMC was lower, whereas cell‐associated IL‐38 expression was comparable. In vitro, IL‐38 is released from CD19^+^ B cells after stimulation with rituximab. Intravenous LPS in humans failed to induce circulating IL‐38, compared to 100‐fold induction of IL‐6 and IL‐1 receptor antagonist. In a cohort of 296 subjects with body mass index > 27 at high risk for cardiovascular disease, IL‐38 plasma concentrations were significantly lower than in healthy subjects (*p* < 0.0001), and lowest in those with metabolic syndrome (*p* < 0.05). IL‐38 also correlated inversely with high sensitivity C‐reactive protein (*p* < 0.01), IL‐6, IL‐1Ra, and leptin (*p* < 0.05). We conclude that a relative deficiency of the B cell product IL‐38 is associated with increased systemic inflammation in aging, cardiovascular and metabolic disease, and is consistent with IL‐38 as an anti‐inflammatory cytokine.

## Graphical abstract

We propose that circulating B cells are a major source of the anti‐inflammatory cytokine interleukin 38 (IL‐38). Circulating IL‐38 concentrations are reduced by old age, being overweight and having metabolic syndrome. In overweight subjects, a relative IL‐38 deficiency is associated with chronic inflammation and increased cardiovascular disease risk.

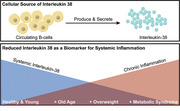

## Introduction

Interleukin 38 (IL1F10) was discovered in silico in 2001, was initially termed IL‐1HY2, and subsequently assigned IL1F10 [1, 2]. The *IL1F10* gene is located on chromosome 2 between two receptor antagonists of the IL‐1 family, *IL*‐*1RN* and *IL*‐*36RN*, with which it shares protein sequence homologies of 41% and 43%, respectively [1].

IL‐38 binds to the IL‐36 receptor (IL‐1R6) and exhibits anti‐inflammatory properties, for example by reducing the *Candida albicans* induced T helper (Th) 17 response in peripheral blood mononuclear cells (PBMC) and by reducing inflammation, IL‐1β, and IL‐6 in murine models of arthritis [3, 4]. Furthermore, IL‐38 can inhibit the induction of IL‐6 and IL‐8 from LPS‐stimulated PBMC and macrophages [5, 6, 7]. In vivo, IL‐38 dampens the Th17 response and attenuates inflammation in murine models of psoriasis, and IL‐38‐deficient mice show an exacerbated Th17 response [8].

Mora et al. demonstrated that recombinant IL‐38 also binds to IL‐1R9 on macrophages to inhibit IL‐6 secretion through restraining JNK induction and reducing AP1 and NFκB activity [6]. Furthermore, IL‐38 can inhibit the IL‐1R9 on γδ‐T cells and thereby reduce the Th17 response in mice [8]. IL‐1R9 is located on the X‐chromosome and crucial for the development of cognition [9]. Boys with mutations in IL‐1R9 suffer from mental retardation and autism [10]. More recently, the function of IL‐38 has gained interest in the context of inflammatory and autoimmune diseases such as rheumatoid arthritis, psoriasis, systemic lupus erythematosus, Crohn's disease, primary Sjögren syndrome, hepatitis, hidradenitis suppurativa and gestational diabetes, along with asthma, retinopathy, ST‐elevated myocardial infarction (STEMI), coronary artery disease, cancer, and sepsis [5, 7, 11, 12, 13, 14, 15, 16, 17, 18, 19, 20, 21]. SNPs associated with *IL1F10* are associated with serum C‐reactive protein (CRP) concentrations in a genome‐wide association study [22], and recombinant IL‐38 inhibits CRP and IL‐1β production by PBMC from patients with hyperlipidemia [20]. Taken together, IL‐38 functions as a suppressor of inflammatory diseases.

To date, little information is available on the cellular source(s) of circulating IL‐38. Also, there are no large‐scale studies on circulating IL‐38 concentrations either in healthy or overweight subjects, or in association with systemic markers of inflammation, particularly in overweight subjects at risk for cardiovascular disease (CVD). Here, we have assessed the source of IL‐38 and its correlations with systemic markers of inflammation.

## Results

### Circulating IL‐38 in healthy subjects

**Figure 1:**
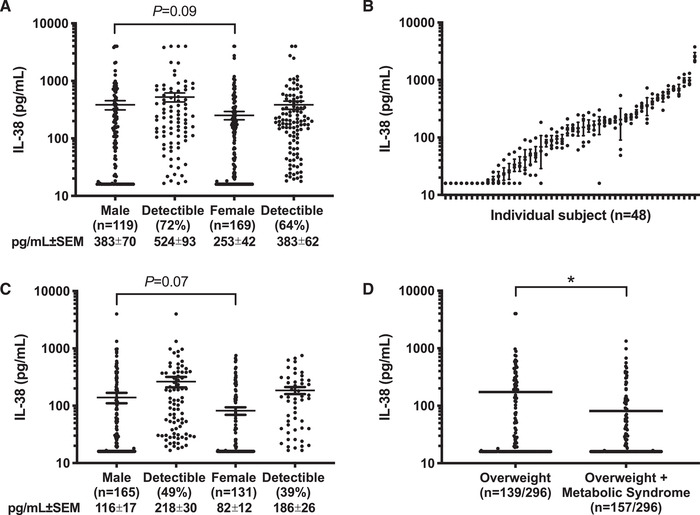
Level of IL‐38 in healthy subjects. (A) Mean ± SEM of plasma IL‐38 concentrations in 119 male and 169 female healthy subjects of the healthy cohort. Shown separately by sex is the percentage of subjects with levels above the lower limit of quantitation (16 pg/mL). (B) Mean ± SEM of plasma IL‐38 concentrations in 48 healthy subjects (61% male) in samples collected at four visits at 3‐month intervals over the course of 1 year. (C) Mean ± SEM of plasma IL‐38 concentrations in 165 male and 131 female of the overweight cohort. Shown separately by sex is the percentage of subjects with levels above the lower limit of quantification (16 pg/mL). (D) Mean ± SEM of plasma IL‐38 concentrations in the overweight cohort divided by metabolic syndrome status. Per cohort, IL‐38 concentrations were measured in one experiment by ELISA. Statistical analysis was performed using the Mann–Whitney test, *p < 0.05.

We measured resting circulating IL‐38 in a cohort of 288 healthy subjects. Plasma IL‐38 concentrations were detectable in 72% of 119 male and in 64% of 169 healthy female subjects (Fig. 1A, *p* = 0.09). As shown in Fig. 1B, IL‐38 concentrations were stable over the course of four seasons in 48 healthy subjects from the healthy cohort, and each sample correlated with the yearly average of that subject (all visits *p* < 0.0001). In addition, IL‐38 concentrations were consistently detectable in four out of four visits for 65% of the subjects and consistently undetectable for 17% of the subjects.

IL‐38 concentrations correlated inversely with age (Spearman *r* = −0.14, 95%CI −0.26 to −0.02, *p* = 0.02). No correlation was found between IL‐38 concentrations and body mass index (BMI). We also found that IL‐38 concentrations did not correlate with circulating IL‐1β, IL‐1Ra, IL‐6, IL‐10, IL‐18, IL‐18BP, TNF‐α, alpha‐1‐antitrypsin (AAT), resistin, leptin, or adiponectin in healthy subjects (Supporting Information Table S1).

### IL‐38 in overweight individuals at risk for cardiovascular disease

We next studied IL‐38 concentrations in a cohort of 296 overweight subjects at risk for cardiovascular events. The subjects were more than 55 years old with a BMI of > 27 kg/m^2^, of which 54% had metabolic syndrome based on the NCEP‐ATP III criteria [23], 54% had carotid artery plaques as determined by ultrasound, and 56% had hypertension [24]. Incomparison, the subjects of the healthy cohort had a mean BMI of 22.2 ± 3. First, we observed that IL‐38 was detectable in 49% of 165 males and in 39% of 131 females, and that there was no significant difference between males and females (*p* = 0.07) (Fig. 1C). In comparison to the healthy cohort, IL‐38 concentrations were on average 3.3‐fold lower in overweight male subjects and 3.1‐fold lower in overweight female subjects (*p* < 0.0001). It should be noted that on average, the overweight cohort was older than the healthy cohort (mean age 29 ± 5 vs. 67 ± 14, *p* < .0001).

Overweight individuals with metabolic syndrome often exhibit a pro‐inflammatory state, in which circulating mediators serve as biomarkers for CVD. As shown in Fig. 1D, overweight individuals with metabolic syndrome have significantly lower concentrations of IL‐38 than overweight individuals without metabolic syndrome (*p* < 0.05). We also observed that IL‐38 concentrations in the overweight cohort correlated negatively with high sensitivity (hs)CRP (*p* < 0.01), leptin, IL‐6, and IL‐1Ra (all *p* < 0.05) (Table 1 and Supporting Information Fig. S1). Also, while IL‐1β concentrations correlated negatively with IL‐38, this relationship did not reach statistical significance (*p* = 0.08). IL‐38 concentrations did not correlate with VEGF, adiponectin, IL‐18BP, IL‐18, resistin, or AAT in overweight subjects.

**Table 1:** Plasma IL‐38 correlates inversely with systemic mediators of inflammation in overweight subjects

Plasma IL‐38 correlation with	Spearman *r* (95% CI)	*p* value
hsCRP	−0.16 (−0.27 to –0.04)	0.008
Leptin	−0.13 (−0.25 to –0.01)	0.024
IL‐6	−0.12 (−0.23 to 0.001)	0.045
IL‐1Ra	−0.12 (−0.23 to 0.003)	0.049
IL‐1β	−0.10 (−0.22 to 0.01)	0.076
VEGF	−0.06 (−0.17 to 0.06)	0.338
Adiponectin	−0.048 (−0.17 to 0.07)	0.414
IL‐18Bp	−0.04 (−0.16 to 0.07)	0.444
IL‐18	−0.04 (−0.16 to 0.08)	0.500
Resistin	−0.03 (−0.15to 0.09)	0.579
Alpha‐1 antitrypsin	0.01 (−0.11 to 0.13)	0.848

Plasma IL‐38 correlates with circulating markers of inflammation in a cohort of 296 overweight individuals at risk for cardiovascular disease. Each cytokine was measured during a single ELISA using aliquotted samples. Shown are the Spearman correlations including the 95% confidence interval and the respective *p* value.

### Circulating IL‐38 concentrations in experimental human endotoxemia

**Figure 2:**
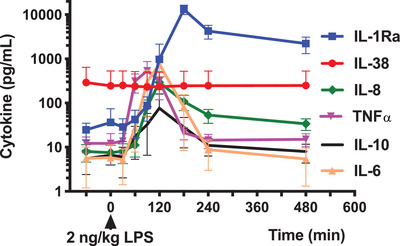
Cytokine analysis in human experimental endotoxemia. Comparison of plasma IL‐38, IL‐1Ra, IL‐8, TNF‐α, IL‐10, and IL‐6 concentrations at indicated time points before and after administration of 2 ng/kg *E. coli* LPS (*T* = 0 minutes) in a model of human experimental endotoxemia in healthy subjects. IL‐38 was measured by ELISA and the other cytokines by a simultaneous Luminex assay. Data from individual experiments (N = 10) were pooled and the data were presented as mean ± SEM.

Human experimental endotoxemia provides a model of sterile systemic inflammation, in which the kinetics of circulating immune mediators can be observed [25]. To investigate the kinetics of IL‐38 in experimental human endotoxemia, plasma IL‐38 concentrations were measured before and after a bolus injection of LPS. As shown in Fig. 2, plasma IL‐38 concentrations did not change after LPS infusion, whereas IL‐1Ra, IL‐8, TNF‐α, IL‐10, and IL‐6 were induced severalfold by LPS. As shown in the Supporting Information Table S2, baseline IL‐38 concentrations did not correlate with the fold increase or area under the curve of LPS‐induced IL‐1Ra, IL‐6, IL‐8, IL‐10, TNF‐α, MIP‐1α, MIP‐1β, and MCP‐1, which were induced by LPS between sevenfold and 497‐fold on average. We conclude that concentrations of IL‐38 neither change nor predict changes of other circulating mediators of inflammation in experimental human endotoxemia.

### Circulating IL‐38 concentration correlates with B cell surface markers

After determining circulating concentrations of IL‐38 in healthy subjects as well as in subjects at risk for CVD, we examined the cellular source of circulating IL‐38. We compared IL‐38 concentrations at the time of blood sampling from healthy subjects at rest to the expression of cell surface markers on circulating cells by flow cytometry. Detectable IL‐38 plasma concentrations and absolute circulating cell counts were available for 190 subjects of the healthy cohort. As shown in Table 2, IL‐38 plasma concentrations correlated positively with absolute numbers of several B cell subsets, most notably memory B cells and plasmablasts (*p* < 0.05). In contrast, IL‐38 plasma concentrations correlated inversely with natural killer T (NKT) cells (*p* < 0.05). The significant correlations are shown in Supporting Information Fig. 2, and a list of all the cell subsets that were studied can be found in Supporting Information Table S3.

**Table 2:** Plasma IL‐38 is associated with several B cell subsets in healthy volunteers

Plasma IL‐38 correlation with	Spearman *r* (95% CI)	*p* value
Plasmablast/cell (IgM+ CD38++ CD27+)	0.21 (0.06 to 0.34)	0.004
IgM only CD27− (IgD− IgM+ CD27−)	0.19 (0.05 to 0.33)	0.007
IgM only (IgD− IgM+)	0.18 (0.04 to 0.32)	0.011
IgM only memory (IgD− IgM+ CD27)	0.15 (0.01 to 0.29)	0.044
Plasmablast (IgD− IgM− CD38++)	0.18 (0.03 to 0.32)	0.014
Plasmablast/cell (CD19+ CD20−)	0.15 (0.007 to 0.29)	0.035
NKT cell (CD3+ CD56+)	−0.17 (−0.31 to −0.02)	0.016

Plasma concentrations of IL‐38 are associated with a B cell phenotype. IL‐38 concentrations in 190 healthy subjects from the healthy cohort were measured by ELISA and associated with immune cell subsets measured by flow cytometry in the same sample. Immune cell subsets were analyzed in freshly obtained samples and IL‐38 concentrations were determined by a single ELISA using aliquotted plasma samples. Shown are the Spearman correlations including the 95% confidence interval and the respective *p* value. Circulating leukocyte data were previously published and re‐analyzed for correlations with plasma IL‐38 concentrations with permission from Aguirre‐Gamboa et al. [42].

### Isolated B cells from PBMC express IL‐38 and produce IL‐38 in vitro

**Figure 3:**
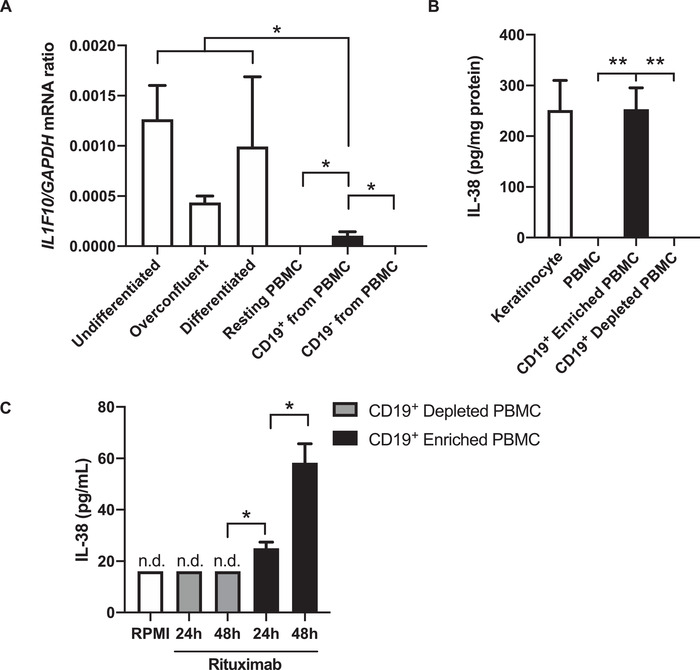
IL‐38 expression in PBMC and keratinocytes. (A) *IL1F10* and *GAPDH* mRNA expression was detected by RT‐qPCR in keratinocyte cultures that were either undifferentiated, overconfluent, or terminally differentiated, as well as resting PBMC isolated from venous blood, isolated CD19^+^ B cells from these PBMC, and the CD19^+^ depleted flow‐through. Expression of *IL1F10* was normalized to *GAPDH* using the 2^−ΔΔCt^ method. (B) IL‐38 protein expression was measured in cell lysates by ELISA and expressed as pg IL‐38 per mg total protein. (C) IL‐38 protein in the supernatant of CD19^+^ enriched or depleted PBMC after 24 h and 48 h stimulation with rituximab and measured by ELISA. Data are expressed as mean ± SEM from three independent experiments with a total of three donors of keratinocytes and PBMC that were analyzed in parallel by RT‐qPCR and ELISA. N.d.: not detected. Unpaired *T* test, *p < 0.05, **p < 0.01.

The gene expression of *IL1F10* has been detected in PBMC of STEMI and hyperlipidemia patients [14, 20]. We next addressed the issue of *IL1F10* expression by PBMC from healthy subjects. We demonstrate that *IL1F10* gene expression was detectable in isolated CD19^+^ B cells from PBMC, while it was not detectable in PBMC depleted of CD19^+^ B cells (Fig. 3A). As a positive control for *IL1F10* expression, we analyzed human keratinocytes that were undifferentiated, overconfluent, or terminally differentiated. The keratinocyte samples expressed *IL1F10* mRNA fourfold to 12‐fold higher than isolated CD19^+^ B cells from PBMC (*p* < 0.05). As shown in Fig. 3B, protein concentrations of IL‐38 were only detectable in lysates from CD19^+^ PBMC and not total PBMC or CD19^+^ depleted PBMC (*p* < 0.01). IL‐38 protein levels were similar in lysates of keratinocytes and isolated B cells, indicating that *IL1F10* mRNA expression does not necessarily correlate with IL‐38 protein concentrations. In the supernatants of CD19^+^ B cells, but not PBMC depleted of CD19^+^ B cells, IL‐38 was present after 24 and 48 h stimulation with rituximab, an anti‐CD20 antibody (Fig. 3C).

## Discussion

This is the first study to examine circulating IL‐38 concentrations in large cohorts of humans during healthy and inflammatory conditions. We measured IL‐38 concentrations in plasma of 288 randomly selected individuals who were part of a larger 500 subject cohort of healthy subjects [26, 27]. IL‐38 concentrations were detectable (> 16 pg/mL) in 72% of men and 64% of women, and although the difference was not statistically significant (*p* = 0.09), the data indicate a trend toward higher concentrations in men. Furthermore, IL‐38 concentrations were stable in a subset of 48 healthy subjects over four seasons. We also compared IL‐38 concentrations with the concentrations of other cytokines and mediators previously measured in this healthy cohort [27]. For example, in these subjects myeloid‐derived IL‐1β, IL‐6, and IL‐1Ra each correlate with one another, and IL‐1β correlate inversely with the hepatic serine protease inhibitor AAT, an endogenous inhibitor of inflammation [27, 28]. However, as a B cell product, IL‐38 did not correlate with circulating IL‐1β, IL‐1Ra, IL‐6, IL‐18, IL‐18BP, VEGF, AAT, resistin, leptin, or adiponectin. We observed that IL‐38 concentrations negatively correlated with age, whereas IL‐6, IL‐1Ra, and IL‐18BP increased with age [27]. BMI positively correlated with plasma concentrations of IL‐1Ra, IL‐18, IL‐6, leptin, and hsCRP [27], but not with IL‐38. We conclude that IL‐38 concentrations do not correlate with myeloid‐derived cytokines in healthy subjects.

Next, we investigated plasma IL‐38 concentrations in a cohort of overweight subjects that are in a state of chronic, low‐grade inflammation, and at increased risk of a cardiovascular event [24]. First, we found that IL‐38 concentrations were significantly lower in overweight individuals compared to healthy volunteers. Relevant to CVD risk, IL‐38 concentrations were lower in subjects with high concentrations of circulating hsCRP. As the Canakinumab Anti‐inflammatory Thrombosis Outcome Study [29] of 10 061 similarly overweight subjects with hsCRP > 2 mg/L has shown that treament with the IL‐1β neutralizing antibody canakinumab reduces CVD, we measured plasma IL‐1β concentrations in the overweight cohort. We observed higher concentrations of IL‐1β in those subjects with low IL‐38, although this correlation did not reach statistical significance. Since in the healthy cohort of 500 subjects IL‐1β concentrations significantly correlated positively with IL‐6 [27], we investigated the relationship between IL‐38 and IL‐6 in the overweight cohort and found a significant inverse correlation. This inverse relationship of IL‐38 with IL‐6 is consistent with an anti‐inflammatory property of IL‐38 [30]. While in healthy subjects IL‐1β and IL‐6 correlate, and IL‐1β is known to induce IL‐6, we hypothesize that IL‐38 functions to may suppress IL‐1β production. Indeed, we previously observed that recombinant IL‐38 suppresses IL‐1β in murine models of experimental arthritis [4].

In the overweight cohort, IL‐38 concentrations were also lower in individuals with high concentrations of leptin. Others have reported that leptin and hsCRP are elevated in individuals with CVD risk and metabolic syndrome [31]. In our study, IL‐38 concentrations were lower in overweight individuals with metabolic syndrome compared to those without metabolic syndrome. Thus, low concentrations of IL‐38 are found in subjects with metabolic syndrome with high hsCRP and leptin.

There have been several reports on the link between IL‐38 and hsCRP. In patients with acute STEMI, IL‐38 plasma concentrations were elevated concurrently with hsCRP concentrations from the time of admission until 7 days later [14]. We hypothesize that chronic low concentrations of IL‐38 in overweight individuals are a biomarker of CVD risk, and that an increase in IL‐38 during an acute cardiovascular event represents a response to limit inflammation and restore homeostasis [14]. In patients with an acute cardiovascular event, IL‐1Ra concentrations are also elevated to limit inflammation and restore homeostasis [32].

These findings are consistent with lower concentrations of IL‐6, hsCRP, and IL‐1β that occur in overweight subjects with higher concentrations of IL‐38, that is, those without metabolic syndrome. In patients with hyperlipidemia, *IL1F10* mRNA in PBMC and IL‐38 in serum were elevated compared to healthy controls [20]. PBMC from patients with hyperlipidemia, when treated in vitro with recombinant IL‐38, reduced gene expression and protein secretion of IL‐6, IL‐1β, and CRP [20]. In that study, patients with high serum concentrations of IL‐38 were more sensitive to lipid‐lowering therapy with atorvastatin [20]. In mice, overexpression of *Il1f10* resulted in reduced hepatic fat accumulation, liver damage in high‐fat diet induced obesityand reduced adipocyte hypertrophy [33]. Together, human and mouse studies support the concept that high concentrations of IL‐38 are functional in suppressing inflammation, while low concentrations are a biomarker for cardiovascular and metabolic risk. In acute disease, plasma IL‐38 correlates with CRP in rheumatoid arthritis patients, and is elevated in comparison to healthy controls [34].

To our knowledge, this is the first study of circulating IL‐38 in a human model of systemic inflammation, in which human volunteers receive a single bolus of intravenous LPS. We observed that IL‐38 concentrations were stable after infusion with LPS, whereas IL‐6, IL‐8, IL‐10, IL‐1Ra, TNF‐α, MIP‐1α, MIP‐1β, and MCP1 each increased several fold, as expected [25]. Plasma concentrations of IL‐38 in this experimental endotoxemia study were comparable to those detected in the cohort of healthy volunteers. Furthermore, baseline IL‐38 concentrations did not predict the fold change or area under the curve of other circulating mediators. This is in contrast with the observations of Xu et al., who demonstrated that IL‐38 concentrations are elevated in serum from adult and pediatric sepsis patients compared to healthy controls [19].

Previous reports show that *IL1F10* mRNA is predominantly present in the keratinocytes of the skin, the spleen, and proliferating B cells of the tonsil [2, 15, 35]. In colon biopsies of inflammatory bowel disease patients, *IL1F10* expression was co‐localized completely with a portion of CD19^+^ B cells, while *IL1F10* expression was not detected in CD68^+^ macrophages or CD3^+^ T cells [35]. Here, we demonstrate that *IL1F10* gene expression is specific for isolated B cells from PBMC, but not total PBMC or the flow‐through that is depleted of B cells. In these B cells, *IL1F10* mRNA expression is fourfold to 12‐fold lower when compared to primary human keratinocytes. Interestingly, B cells can also express IL‐36 family members IL‐36α [36] and IL‐36γ [37] and their receptor IL‐36R [38], and hence the additional production of the IL‐36R inhibitor IL‐38 by B cells may bring balance to IL‐36 signaling.

After demonstrating that *IL1F10* expression is specific to B cells isolated from PBMC, we sought to relate plasma IL‐38 concentrations in healthy subjects to the cell subsets that are present in the same blood sample. In 190 healthy subjects, circulating concentrations of IL‐38 correlated with several B‐cell subsets, most notably memory B cells and plasmablasts. We observed that circulating B cell numbers and IL‐38 concentrations both decline with age. In fact, of the four large groups of circulating B cell subsets (immature‐, naïve, memory‐B lymphocytes, and plasma cells), only memory B lymphocytes and plasmablasts/cells decrease with age, both in relative and absolute counts [39]. The single negative correlation between IL‐38 and circulating cell subsets was with NKT cells. NKT cells generally become activated in pro‐inflammatory conditions, further underscoring the anti‐inflammatory nature of IL‐38 [40]. Noteworthy is that there was no correlation between IL‐38 concentrations and circulating neutrophils, monocytes, or T cells.

We demonstrate that IL‐38 is a B cell derived cytokine in healthy subjects, which is inversely associated with markers of innate inflammation that are associated with an increased risk for cardiovascular disease in overweight subjects. Furthermore, a relative IL‐38 deficiency arises with older age, and a clear reduction of circulating IL‐38 is observed in subjects that are overweight, while IL‐38 concentrations are lowest in overweight subjects with metabolic syndrome. Future studies on the relationship between IL‐38 and the separate components of the metabolic syndrome would further strengthen these findings.

## Materials and methods

### Subjects

Participants received detailed printed and oral information and gave written consent. Inclusion of subjects and experiments were conducted according to the principles expressed in the Declaration of Helsinki. The healthy Dutch 500FG cohort was approved by the Ethical Committee of Radboud University Medical Center (NL42561.091.12, 2012/550), as well as the 300 Overweight study (NL34462.091.10). The “Remote Ischemic Preconditioning on Inflammation During Human Endotoxemia” (RISPENDO) trial was approved (NL53584.091.15). Plasma and clinical data in the present study were derived from the healthy cohort [27], the overweight cohort [24], and the human endotoxemia trial [41]. The studies on healthy and overweight subjects are part of the Human Functional Genomics Project (HFGP) (www.humanfunctionalgenomics.org).

### Healthy subjects

The healthy cohort consists 534 healthy individuals of Caucasian origin aged > 18 years, sampled between July 2013 and December 2014 at the Radboud University Medical Center, The Netherlands [26, 27, 42]. Exclusion criteria were pregnancy/breastfeeding, chronic or acute disease at the time of sampling, and medication use, either prescribed or over the counter, in the last month before the study. The median age was 23 years (interquartile range [IQR] 21–27 years) and 56% were female. The median BMI was 22.2 (20.7–24.4) and 83.1% had a BMI below 25. 48% of women used an oral contraceptive, and 13.2% of subjects regularly used tobacco. From the healthy cohort, 288 plasma samples were available for measurement of plasma IL‐38 concentrations.

### Overweight individuals

The 300 overweight cohort [24] consists of individuals aged 55–80 of European origin with a BMI > 27. Subjects with a recent cardiovascular event (myocardial ischemia, transient ischemic attack, stroke <6 months), a history of bariatric surgery or bowel resection, inflammatory bowel disease, renal dysfunction, increased bleeding tendency, use of oral or subcutaneous anti‐coagulant therapy, use of thrombocyte aggregation inhibitors other than acetylsalicylic acid, and carbasalate calcium were excluded. The presence of metabolic syndrome was diagnosed using the clinical criteria defined in the National Cholesterol Education Program (NCEP‐ATP III criteria). The median age was 67 years (IQR 63–71 years) and 45% were female. The median BMI was 29.9 (28.3–31.9). None of the women used an oral contraceptive and 8.6% of subjects regularly used tobacco. From the 300 overweight cohort, 296 plasma samples were available for measurement of IL‐38 concentrations.

### Experimental human endotoxemia

In the RISPENDO trial control group, ten healthy male subjects were included, aged 22.1 ± 3.1 years, 183 ± 11 cm, weighing 73 ± 7.8 kg, and having a BMI of 21.8 ± 1.8. To achieve a controlled inflammatory state, subjects were pre‐hydrated and subsequently infused with 2 ng/kg *Escherischia coli* LPS intravenously in 1 min at *t* = 0 h. Blood was collected in EDTA before LPS infusion (*t* = −60 and 0), and after 30, 60, 90, 120, 240, and 480 min.

### Blood collection and plasma preparation

Venous blood from the healthy, overweight, and RISPENDO cohorts was collected in EDTA tubes and centrifuged immediately at 2000 x *g* for 10 min at 4°C after which the plasma was aliquoted into smaller volumes and stored at −80°C until analysis.

### CD19^+^ cell isolation, keratinocyte culture, and IL‐38 qPCR

Isolation of PBMC was performed as described before [43]. B cells were isolated from PBMCs using MACS CD19 positive isolation kit (Miltenyi Biotech) according the manufacturer's protocol, stained for CD45 and CD19, and > 95% purity was confirmed by flow cytometry. From PBMC, isolated CD19^+^ B cells, and PBMC depleted in CD19^+^ B cells, mRNA and protein were obtained as described below, and IL‐38 expression was detected by RT‐qPCR and ELISA. In addition, CD19^+^‐enriched and CD19^+^‐depleted PBMC were cultured at 100 000 cells/well in 200 μL RPMI supplemented with pyruvate (0.02 mM), glutamax (2 mM), penicillin (100 U/mL), streptomycin (100 μg/mL; all from Gibco, UK), and 10% heat‐inactivated pooled human serum [44]. For stimulation experiments, the cells were cultured with 5 μg/mL rituximab (Roche, Germany), an anti‐CD20 monoclonal antibody, and IL‐38 concentrations were measured by ELISA after 24h and 48h. Human epidermal keratinocytes (Thermo Fisher, Waltham, MA) were cultured in Epilife medium (Thermo Fisher) supplemented with human keratinocyte growth supplement (Thermo Fisher). Keratinocytes were plated at 1.0 × 10^5^ cells/well in 12‐well plate. Undifferentiated keratinocytes were harvested at 100% confluency. Overconfluent keratinocyte harvested 4 days post 100% confluency. Starting at 90% confluency, differentiated keratinocytes were generated by first 24 h culture in 1.0 mM Ca^2+^, followed by 72 h culture in 1.5 mM Ca^2+^. mRNA and protein were obtained as described below, and IL‐38 expression was detected by RT‐qPCR and ELISA.

### RNA isolation and RT‐qPCR

Total RNA from keratinocytes and PBMC was extracted using an RNeasy Mini Kit (QIAGEN, Venlo, The Netherlands). cDNA synthesis was performed with iScript cDNA Synthesis Kit (BioRad, Hercules, CA). cDNA was synthesized as follows: 5 min at 25°C, 20 min at 46°C, and then 1 min at 95°C. PCR amplification and quantitation were performed using a Power Up SYBR Green PCR Master Mix (Applied Biosystems, Foster City, CA) on the AriaMx Real‐Time PCR system (Agilent Technologies). PCR was performed as follows: one cycle 2 min at 50°C, followed by 5 min at 95°C: 40 cycles 5 min at 95°C; gene‐specific annealing temperature 1 min at 60°C: and then 30 s at 95°C, followed by 30 s at 65°C and 30 s at 95°C. Primer sequences for IL‐38 are similar to those used in [15]: forward—5′‐AAG GTC CCC ATT TTC CTG GG‐3′; reverse—5′‐CTC AAT GTT CAC ATC CTC CAG‐3′. GAPDH: forward—5′‐TGC ACC ACC AAC TGC TTA GC‐3′; reverse—5′‐GGC ATG GAC TGT GGT CAT GAG‐3′. Expression of *IL1F10* was calculated as a ratio to GAPDH using the 2^−ΔΔCt^ method. In addition, keratinocyte and PBMC were lysed in RIPA buffer (Sigma) supplemented with protease inhibitors (Roche), and centrifuged at 13 000 x *g* for 20 min at 4°C. Protein concentrations of the cleared supernatants were measured by Bio‐Rad protein assay (Bio‐Rad Laboratories, Hercules, CA). Cell‐associated IL‐38 concentrations were measured by ELISA and expressed as pg/mg total protein.

### Cytokine measurements

The human IL‐38/IL1F10 DuoSet ELISA (BioTechne, Minneapolis, MN, USA) [19, 45] was used according to the manufacturer's instructions, with the exception that the sample was incubated with the capture antibody in the ELISA plate overnight at 4°C. Plasma samples and cell culture supernatants were diluted 1:1 in PBS containing 1% BSA (Sigma). A standard curve of 8–2000 pg/mL yielded a lower limit of quantification of 16 pg/mL and an upper limit of quantification of 4000 pg/mL. Values below the detection limit were assigned the lower limit of quantification. To investigate the recovery of IL‐38 in the IL‐38 Elisa, we added 200 pg/mL IL‐38 to plasma and compared this to IL‐38 added to PBS alone. Approximately 55% of the IL‐38 that was added to the plasma was detected, indicating that plasma factors may influence the detection of IL‐38 protein. In the healthy cohort, the circulating mediators resistin, leptin, adiponectin, CRP, and AAT were measured in EDTA plasma using DuoSet ELISA (BioTechne) following the manufacturer's protocol. Plasma IL‐1β, IL‐6, IL‐18, VEGF, TNF‐α, and IL‐10 were measured in Simple Plex cartridges using the ELLA apparatus (Protein Simple, San Jose, CA). Vitamin D levels were analyzed by LC‐MS as described previously [27]. In the overweight cohort, AAT, hsCRP, adiponectin, and resistin were measured by DuoSet ELISA (BioTechne). IL‐1β and IL‐18 were measured using Simple Plex cartridges. In the healthy and overweight cohorts, IL‐1Ra and IL‐18 binding protein were measured by Quantikine ELISA (BioTechne). In samples of the human endotoxemia study, TNF‐α, IL‐1Ra, IL‐6, IL‐8, IL‐10, MIP‐1β, MIP‐1α, and MCP‐1 were measured using a simultaneous Luminex assay according to the manufacturer's instructions (Millipore, Bellerica, MA, USA).

### Flow cytometry of circulating cell subsets

The data on circulating leukocyte subsets were previously published, and correlated in this study to plasma IL‐38 concentrations with permission from Aguirre‐Gamboa et al. [42]. In short, flow cytometry was performed within 2–3 h after sample collection on a 10‐color Navios flow cytometer (Beckman Coulter, Fullerton, CA) equipped with three solid‐state lasers (488, 638, and 405 nm). Supporting Information Table S3 contains the list of the CD markers. The purity of CD19^+^ B cells isolated from PBMC was confirmed with antibodies to CD19 and CD45. Data were analyzed using Kaluza software version 1.3 (Beckman Coulter).

### Statistical analysis

Correlations and corresponding *p*‐values were calculated using the Pearson or the rank‐based Spearman correlation using Graphpad Prism software v7.0 (GraphPad Software). When two parameters were compared, the rank‐based Mann–Whitney test was used unless otherwise indicated. All statistical tests were two sided, and *p* values < 0.05 were considered significant.

## Conflict of interest

The authors declare no commercial or financial conflict of interests.

## Author contributions

DMG, LABJ, and CAD conceived experiments and wrote the paper. DMG, MJ, ICLM, RH, JZ, MK, JHWH, NPR, MF, SM, FLV, LABJ, MGN, JG, and MK designed experiments. DMG, MJ, ICLM, KS, TY, LM, and JZ performed experiments.

### Peer review

The peer review history for this article is available at https://publons.com/publon/10.1002/eji.201948390.

**Abbreviations**
AAT: alpha‐1‐antitrypsin
BMI: body mass index
CVD: cardiovascular disease
CRP: C‐reactive protein
hs: high sensitivity hs
IL: Interleukin
IQR: Interquartile range
NKT: Natural Killer T
PBMC: peripheral blood mononuclear cells
STEMI: ST‐elevated myocardial infarction
Th: T helper

## Supporting information

Supporting Information

## Data Availability

Data are available upon reasonable request from the authors.
