# Supplementary material for: Reduced concentrations of the B cell cytokine interleukin 38 are associated with cardiovascular disease risk in overweight subjects
Source: Eur J Immunol. 2020 Nov 19;51(3):662–71. doi: 10.1002/eji.201948390 (PMC7983920; doi:10.1002/eji.201948390)
Supplement: Supplementary file 1 — Supporting Information [file EJI-51-662-s001.docx]

Supporting Information to:

Reduced concentrations of the B cell cytokine Interleukin 38 are associated with cardiovascular disease risk in overweight subjects.

Dennis M. de Graaf, Martin Jaeger, Inge C. L. van den Munckhof, Rob ter Horst, Kiki Schraa, Jelle Zwaag, Matthijs Kox, Mayumi Fujita, Takeshi Yamauchi, Laura Mercurio, Stefania Madonna, Joost H.W. Rutten, Jacqueline de Graaf, Niels P. Riksen, Frank L. van de Veerdonk, Mihai G. Netea, Leo A.B. Joosten and Charles A. Dinarello

Supporting Information Figure 1.

**Supporting Information Figure 1.** Correlation of plasma IL‑38 levels with markers of inflammation in overweight individuals. Plasma IL‑38 levels were associated with circulating factors hsCRP, leptin, IL‑6, IL‑1Ra, and IL‑1β. Each dot represents one individual of the overweight cohort. N=296.

Supporting Information Figure 2.

**Supporting Information Figure 2.** Correlation of plasma IL‑38 levels in healthy volunteers with circulating cell subsets. Each dot represents one individual of the healthy Dutch Cohort. N=190. Circulating leukocyte data was previously published by Aguirre-Gamboa et al. [1] and re-analyzed with permission for correlations with plasma IL‑38 concentrations.

Supporting Information Table I.

Plasma IL-38 does not correlate with systemic mediators of inflammation in healthy subjects

| Healthy Volunteers | Spearman r (95% CI) | *P* value | n |
| --- | --- | --- | --- |
| IL-1α | 0.04 (-0,16 to 0,22) | 0.72 | 112 |
| IL-1β | 0.06 (-0,06 to 0,19) | 0.31 | 251 |
| IL-1Ra | -0.03 (-0,15 to 0,09) | 0.66 | 286 |
| IL-6 | 0.01 (-0,12 to 0,13) | 0.89 | 251 |
| IL-10 | 0.05 (-0,15 to 0,24) | 0.64 | 110 |
| IL-18 | 0.02 (-0,10 to 0,15) | 0.71 | 251 |
| IL-18BP | 0.04 (-0,08 to 0,16) | 0.48 | 285 |
| TNF-α | 0.10 (-0,10 to 0,28) | 0.31 | 110 |
| AAT | -0.03 (-0,15 to 0,09) | 0.57 | 285 |
| Resistin | 0.06 (-0,06 to 0,17) | 0.35 | 285 |
| Leptin | -0.06 (-0,18 to 0,06) | 0.31 | 285 |
| Adiponectin | -0.04 (-0,16 to 0,08) | 0.46 | 285 |
| VEGF A | -0.02 (-0,15 to 0,10) | 0.71 | 250 |
| Vitamin D | -0.01 (-0,13 to 0,11) | 0.80 | 285 |

**Supporting Information Table I.** Association of plasma IL‑38 concentration with plasma cytokine concentrations in healthy volunteers.

Supporting Information Table II.

Cytokine induction in human experimental endotoxemia

| Cytokine | Fold change | IL-38 correlation to Fold change | | IL-38 correlation to AUC | |
| --- | --- | --- | --- | --- | --- |
|  |  | Spearman r (95% CI) | *P value* | Spearman r (95% CI) | *P value* |
| IL-1Ra | 497 | -0.18 | 0.61 | -0.14 | 0.7 |
| IL-6 | 164 | 0.05 | 0.9 | 0.05 | 0.9 |
| IL-8 | 46 | -0.05 | 0.9 | -0.07 | 0.86 |
| IL-10 | 15 | -0.36 | 0.3 | -0.15 | 0.69 |
| TNFα | 75 | 0.09 | 0.82 | 0.09 | 0.82 |
| MIP-1α | 7 | -0.43 | 0.22 | -0.29 | 0.42 |
| MIP-1β | 100 | 0.12 | 0.74 | 0.1 | 0.79 |
| MCP-1 | 84 | 0 | 1 | -0.17 | 0.64 |

**Supporting Information Table II.** Induction of plasma cytokines in a model of human experimental endotoxemia induced by 2 ng/kg *E. coli* LPS. AUC: Area under the curve.

Supporting Information Table III.

List of CD markers

| Cell subset/Markers | Cell subset/Markers |
| --- | --- |
| B cells (CD19+) | CD8 RA-127- |
| B cells CD3- CD19+ | CD8 RA-127+ |
| CD19+ CD20- Plasma blasts/cells | CD8 RA+127- |
| CD19+ CD20+ B cells | CD8 RA+127+ |
| CD24+ CD38+ | CD8+ CM CD45RA- CD27+ |
| CD24+ CD38+ CD27+ IgM+ | CD8+ CM CD45RO+ CD27+ |
| CD27- IgM+ | CD8+ Eff CD45RA+ CD27- |
| Class non switched memory (IgM+ CD38+ CD27+) | CD8+ Eff CD45RO- CD27- |
| Class switched memory (IgD- IgM- CD38+ CD27+) | CD8+ EM CD45RA- CD27- |
| Classical monocytes (CD14++CD16-) | CD8+ EM CD45RO+ CD27- |
| IgD- CD5+ | CD8+ Naive CD45RA+ CD27+ |
| IgD- CD5++ | CD8+ Naive CD45RO- CD27+ |
| IgD- IgM- | CD8+ T cells |
| IgD- IgM- CD27- | CD8+ T cells |
| IgD- IgM+ | DN (CD4- CD8-) |
| IgD- IgM+ CD27- | DP (CD4+ CD8+) |
| IgD+ CD5+ | Intermediate monocytes (CD14+CD16+) |
| IgD+ CD5++ | Lymphocytes |
| IgD+ IgM- | Lymphocytes |
| IgD+ IgM+ | Lymphocytes |
| IgM only memory (IgD- IgM+ CD27) | Mature naive CD24+ CD38+ |
| IgM- | Monocytes (CD14+) |
| IgM+ CD27- | Natural effector (CD24+ CD38+ IgD+ IgM+) |
| IgM+ CD38++ CD27+ | Neutrophils |
| Memory B cells (IgD+ IgM+ CD27+) | NK (CD56+ CD16-) |
| Naive B cells (IgD+ IgM+ CD27-) | NK bright (CD56++ CD16-) |
| Plasmablast (IgD- IgM- CD38++) | NK cells (CD3- CD56+) |
| Transitional B cell CD27- IgM+ CD24+ CD38high | NK dim (CD56+ CD16+) |
| Transitional B cells (CD24++ CD38++) | NKT cells (CD3+ CD56+) |
| CD4-CD8- | Non-classical monocytes (CD14++CD16+) |
| CD4-CD8+ | Prol CD4+ Tconv |
| CD4+ CD25high Treg | Prol CD4+ Treg |
| CD4+ CM CD45RA- CD27+ | Prol CD8 |
| Cell subset/Markers | Cell subset/Markers |
| CD4+ CM CD45RO+ CD27+ | Prol DN(CD4-CD8-) |
| CD4+ Eff CD45RA+ CD27- | Prol DP(CD4+CD8+) |
| CD4+ Eff CD45RO- CD27- | T cells (CD3+ CD56-) |
| CD4+ EM CD45RA- CD27- | T cells (CD3+ CD56-) |
| CD4+ EM CD45RO+ CD27- | Tconv CD25- CD127+ |
| CD4+ Naive CD45RA+ CD27+ | Treg CD25+ CD127lo/neg |
| CD4+ Naive CD45RO- CD27+ | Treg CD25+ CD127low |
| CD4+ T cells | Treg CD45RA- |
| CD4+ T cells | Treg CD45RA+ |
| CD4+CD8- | Treg FOXP3+ Helios- |
| CD4+CD8+ | Treg FOXP3+ Helios+ |
| CD45RO- CD45RA- T cells | Treg HLA-DR+ |
| CD45RO- CD45RA+ T cells |  |
| CD45RO+ CD45RA- T cells |  |
| CD45RO+ CD45RA+ T cells |  |

**Supporting Information Table III.** List of CD markers detected by flow cytometry of circulating cell subsets used in the 500FG study. Circulating leukocyte data was previously published and re-analyzed with permission from Aguirre-Gamboa et al. [1].

1 **Aguirre-Gamboa, R., Joosten, I., Urbano, P. C. M., van der Molen, R. G., van Rijssen, E., van Cranenbroek, B., Oosting, M., Smeekens, S., Jaeger, M., Zorro, M., Withoff, S., van Herwaarden, A. E., Sweep, F., Netea, R. T., Swertz, M. A., Franke, L., Xavier, R. J., Joosten, L. A. B., Netea, M. G., Wijmenga, C., Kumar, V., Li, Y. and Koenen, H.,** Differential Effects of Environmental and Genetic Factors on T and B Cell Immune Traits. *Cell Rep* 2016. **17**: 2474-2487.
